# Identification of Intrinsic Friction and Torque Ripple for a Robotic Joint with Integrated Torque Sensors with Application to Wheel-Bearing Characterization

**DOI:** 10.3390/s24237465

**Published:** 2024-11-22

**Authors:** Sri Harsha Turlapati, Van Pho Nguyen, Juhi Gurnani, Mohammad Zaidi Bin Ariffin, Sreekanth Kana, Alvin Hong Yee Wong, Boon Siew Han, Domenico Campolo

**Affiliations:** 1 School of Mechanical and Aerospace Engineering, Nanyang Technological University, Singapore 639798, Singapore; 2 Schaeffler Hub for Advanced Research, Nanyang Technological University, Singapore 639798, Singapore

**Keywords:** intrinsic dynamics, linear regression, contact-rich, wheel-bearing inspection

## Abstract

Although integrated joint torque sensors in robots dispel the need for external force/torque sensors at the wrist to measure interactions, an inherent challenge is that they also measure the robot’s intrinsic dynamics. This is especially problematic for delicate robot manipulation tasks, where interaction forces may be comparable to the robot intrinsic dynamics. Therefore, the intrinsic dynamics must first be experimentally estimated under no-load conditions, when the measurement only consists of torques due to the transmission of the robot actuator, before external interactions may be measured. In this work, we propose an approach for identifying and predicting the intrinsic dynamics using linear regression with non-linear radial basis functions. Then, we validate this regression on a wheel-bearing turning task, in which its friction is a measure of quality, and thus must be accurately measured. The results showed that the bearing torque measured by the joint 7 torque sensor was within an RMS error of 11% of the torque measured by the external force/torque sensor. This error is much lower than that before our proposed model in compensating the intrinsic dynamics of the robot arm.

## 1. Introduction

The use of collaborative robots (cobots) has become increasingly prevalent in industrial work settings due to their ability to work closely and interact with humans [1,2,3]. These robots are typically equipped with joint torque sensors, which offer advantages in addressing safety concerns during physical interaction with humans. However, a major drawback with this arrangement is that the joint torque sensors measure the intrinsic dynamics of the robot, including torques from the power transmission system (refer Section 2.1 [4]), as well as the robot’s rigid body dynamics [5,6]. This can lead to measurement inaccuracies, particularly when performing contact tasks, such as inspections that involve rotary motions, where the intrinsic dynamic torques can be comparable to the torques of interaction. In addition, calibration errors and ripple torques from gear motion can also contribute to measurement inaccuracies. Hence, enhancing the accuracy of torque measurement in the robot joints via eliminating the impact of the intrinsic dynamics is necessary.

Modern cobot joints commonly comprise a motor connected in series with a transmission drive, joint torque sensor, encoder and other components such as bearings [7,8]. Harmonic drives have high gear-reduction ratios, which enable higher torque transmission in robot joints within a compact geometry [9]. They consist of three main components, namely, the wave-generator, flexspline and the outer circular spline. The elliptical wave generator causes a periodic deformation of the flexspline when the harmonic drive is operational. This periodic motion results in a periodic noise that has much greater signal amplitude than the electrical noise and is known as torque ripple. It becomes coupled with the output of the torque sensor embedded in the robot joint thereby affecting its measurement [10]. Similarly, inertial and frictional torques or errors in force/torque sensor calibration may also affect the torque sensing performance in robot joints. Accurate torque sensing is vital for many robotic applications, which makes it important to filter out such disturbances in the sensed values. Several methods have been proposed so far to compensate for the intrinsic dynamics of harmonic drives.

### 1.1. Related Work

Common torque ripple compensation techniques include use of Kalman filtering-based methods [11]. Ref. [12] also proposed a redundant adaptive robust Kalman filter (RARKF) for torque estimation that was not just tolerant of modeling error but, also enabled a dynamic balance between optimality and robustness due to load variation on a robot joint by designing it as a function of motor current to deal with modeling error and other disturbances. The harmonic drive kinematic model incorporated the effects of torsional compliance (which also captured hysteresis), non-linear friction as well as the kinematic errors due to machining [13]. The harmonic drive compliance model was incorporated in the torque estimation technique proposed in [14], as well as in [15] but along with unscented Kalman filter (UKF) optimization to improve the accuracy. They found that the estimated torques obtained using the UKF-based optimization algorithm were 54.5% more accurate as compared to the method involving only on the harmonic drive compliance model. Another study by [16] performed a harmonic drive characterization to control torque ripple for vibration control of a flexible robotic system. Two types of controllers were developed for this purpose but only the one based on feed-forward control performed well during actual testing. This also required precise placement of the position sensor at the output. Ref. [10] worked on the disturbance elimination of a modular joint torque sensor in a collaborative robot by considering the two major factors affecting torque measurement: torque ripple and temperature drift due to heating of motor. For torque ripple configuration, they developed a strain gauge configuration based on specified phase difference while for temperature compensation, a Wheatstone bridge was combined with a non-linear temperature drift model.

Motivated to reduce the impact of ripple torque causing errors in sensed torque of the robot arm with multiple joints, we propose a novel model-based method for the compensation of the robot intrinsic dynamics. We identify the different sources of torque measurements in a transmission drive, i.e., calibration offsets in the torque sensor, friction, torque ripple due to gear-teeth interaction and finally the inertia of the system. In this work, we use linear regression with a set of non-linear radial basis functions. To perform a meaningful regression, each of the radial basis functions is chosen to represent one of the contributing factors to the intrinsic dynamics.

### 1.2. Wheel-Bearing Inspection

To validate our approach on an actual industrial problem, the inspection of a rolling wheel-bearing was performed. Typically, wheel-bearing inspections in a factory line constitute a human operator identifying a faulty bearing by turning and sensing the friction. wheel-bearings with greater friction lead to safety concerns due to heat generation and mechanical wear and tear. This in turn leads to reduction in efficiency, increase in noise and in the long term compromises equipment integrity. To automate the quality inspection of such wheel-bearings, it is important to identify the friction offered by the bearing correctly, despite the inherent robot joint intrinsic dynamics. For experimental analyses, we chose a 7DoF Kinova Gen3 ultra-lightweight robot (Kinova, Boisbriand, QC, Canada) with inbuilt joint torque sensors, as the robotic platform. First, the intrinsic dynamics of the robot end-effector are estimated from sensed torque measurements when the joint was driven with no load attached to it—since it will only sense the torques due to the transmission. Then, the robot was commanded using impedance control to grasp the wheel-bearing with a compliant finger-pad and turn it. The predicted intrinsic dynamic torques were subtracted from the torque measurements to identify the torque due to wheel-bearing friction.

This paper is organized as follows; the robot system with torque sensing and its intrinsic dynamics of the actuators are presented in Section 2. The experimental setup is introduced in Section 3, considering both no external load rotation and wheel-bearing rotation. These results are discussed in Section 4, which evaluates the validation with external load cell and repeatability of the proposed methodology. Finally, Section 5 concludes the paper and outlines perspectives for future work.

## 2. Sources of Torque Measurements in a Transmission System

Conventionally, the rigid dynamic model of a robot [4,17,18] with rigid links and infinitely stiff joints is defined as:

(1) $$M\left(q\right)\ddot{q}+C\left(q,\dot{q}\right)\dot{q}+G\left(q\right)=\tau_{rb}$$

where M is the inertia matrix, C captures the Coriolis and centrifugal forces and G accounts for gravity effects, all of which sum up to yield the rigid body dynamics of the robot $\tau_{rb}$. In the presence of external contact and intrinsic dynamical effects (such as friction, damping, etc.), additional terms contribute to the sensed torque $\tau_{s}$ at the joints:

(2) $$\tau_{s}=\tau_{rb}+\tau_{intr}+\tau_{ext}$$

where $\tau_{intr}$ is the intrinsic dynamic torques, $\tau_{ext}=J^{T}W_{ext}$ is the torque due to external interaction with J the robot Jacobian and $W_{ext}$ the wrench due to the external interaction at the end-effector. In this work, we simplify the above to a single joint and thus eliminate the rigid-body dynamical effects, i.e., $\tau_{rb}=0$, but note that the effect of $\tau_{intr}$ will still remain even for a single joint when measuring external interaction. Thus, the goal of this section is to model $\tau_{intr}$ for a single joint assuming no external interaction $W_{ext}$ is present. Once the model is ready for use, we can compute the torque due to the external interaction simply by $\tau_{ext}=\tau_{s}-\tau_{intr}$.

**Figure 1:**
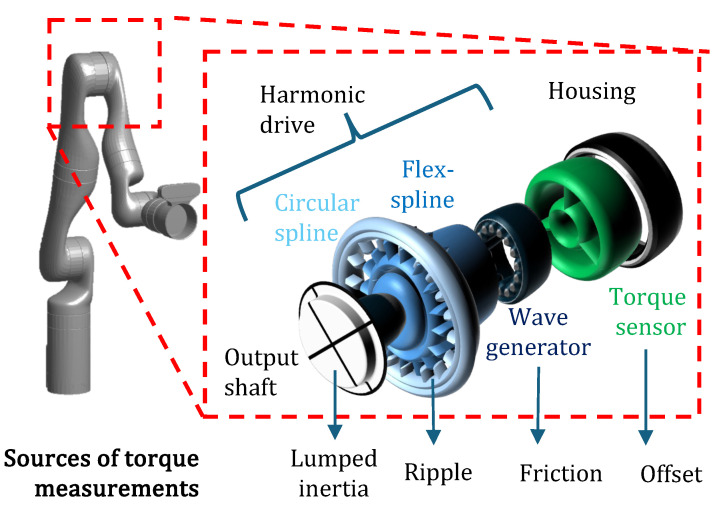
Structure of a joint in a collaborative robot with the location of torque sensor before gearbox. For a comprehensive study, see [5].

Regardless of the sensor placement with regard to the rotor and gearbox assembly, in this work we assume algebraic dependency of the sensed torques on positions, velocities and accelerations. In other words, under no-load conditions (when the robot actuator is freely rotating with no load attached to the output shaft) the sensor reads the torque due to the transmission. For any transmission system, a driving element with a gearbox is connected in series with the torque sensor. The input element of a typical harmonic transmission (see Figure 1) is the wave generator, which is constructed as a hub with an elliptical shape fitted within a wall bearing. This input element can be a source of friction primarily due to the bearings, which we assume to be a sum of (i) Coulomb and (ii) viscous friction. The wave generator is inserted into the rotation-stiff and radially compliant flexspline component, which conforms to the shape of the wave generator. The flexspline has external gear-teeth that engage with the circular spline. The circular spline is a rigid loop with internal gear-teeth. This gear-teeth contact produces periodic (and predictable) torque ripples. We assume these can be modeled as harmonics of the actuator rotational position in this work. The torque sensor itself may have calibration offsets that need to be accounted for in our model. From here onwards, we call these torques as ‘robot intrinsic dynamics’. Finally, the robot/actuator inertia also contributes to the sensed torque. Therefore, in contacts tasks where the accurate determination of forces of interaction with the environment is crucial, it is important that the effects of the rigid body dynamics as well as the aforementioned intrinsic dynamics are compensated for.

An important and well-studied source of torque measurements is due to rigid body motion of the robot body itself. The inverse dynamics model [17,18] accounts for this with inertial, Coriolis and gravity terms. However, in practice, the torque due to intrinsic dynamics and external interaction must be estimated experimentally. It is ideal to devise an experimental protocol where measurements without any external interaction torques are first obtained to estimate the intrinsic dynamics. Following this, any torque due to external interactions may be estimated by compensating for these intrinsic dynamics.

### 2.1. Intrinsic Dynamics of Robot Actuators

In this subsection, we estimate the intrinsic dynamics via linear regression with the help of a set of non-linear radial basis functions. Each of these basis functions represents one of the contributing factors of the intrinsic dynamics. Consider basis functions $\phi_{m}:R\to R,\;\forall \;m\;\in \left[1,\;M\right]$ mapping kinematics states $x:={\left[\theta ,\;\dot{\theta },\;\ddot{\theta }\right]}^{T}$ into scalar torques τ per each joint. Given a set of measurements ${\left\{\tau_{n},x_{n}\right\}}_{n=1}^{N}$, we seek to determine a vector of *M* parameters $w={\left[w_{1},\ldots ,w_{M}\right]}^{T}$ that best fits the target data (in this case torques) $y={\left[y_{1}.\ldots ,y_{N}\right]}^{T}\equiv {\left[\tau_{1}.\ldots ,\tau_{N}\right]}^{T}$, i.e.,

(3) y=Φw+ϵ

where ϵ is a vector of residuals and Φ is the design matrix, defined as:

(4) $$\Phi :=\left(\begin{array}{ccc} \phi_{1}\left(x_{1}\right) & \ldots & \phi_{M}\left(x_{1}\right)\\ \vdots & \vdots & \vdots \\ \phi_{1}\left(x_{N}\right) & \ldots & \phi_{M}\left(x_{N}\right) \end{array}\right)$$

Assuming a full rank design matrix, i.e., rank(Φ)=M<N, the least-squares solution $w^{*}$ to the over-determined linear problem is:

(5) $$w^{*}={\left(\Phi^{T}\Phi \right)}^{-1}\;\Phi^{T}\;y$$

Once the parameters have been determined, then for a new robot state x, the intrinsic dynamics y can be predicted as:

(6) $$y=\sum_{m=1}^{M}w_{m}^{*}\;\phi_{m}\left(x\right)=\left[\phi_{1}\left(x\right)\;\ldots \;\phi_{M}\left(x\right)\right]\;w^{*}$$

#### Selection of Basis Functions

To perform a meaningful regression, we choose the basis functions such that each of them represents one of the contributing factors to the intrinsic dynamics.

*(a)*  *Friction*

There are several models [19] in the literature to model various types of friction. In this work, we choose the simplest, i.e., Coulomb’s friction to model the non-linear behavior of static friction and viscous friction to model the velocity-dependent nature of friction. The total torque due to friction $\tau_{f}$ is written as their sum:

(7) $$\tau_{f}=\phi_{v}w_{v}+\phi_{c}^{+}w_{c}^{+}+\phi_{c}^{-}w_{c}^{-}$$

where $\phi_{v}$ represents the basis function for the viscous friction and $\phi_{c}^{+},\phi_{c}^{-}$ represent the basis functions for the positive and negative Coulomb frictions. Here, $w_{v},w_{c}^{+},w_{c}^{-}$ represent parameters that best fit these basis functions to the target torque data.

Specifically, the Coulomb friction [20] is given by:

(8) $$\tau_{c}=\left\{\begin{array}{c} \phi_{c}^{+}w_{c}^{+}\;if\;\dot{\theta }>0\\ 0\;\;\;if\;\dot{\theta }=0\\ \phi_{c}^{-}w_{c}^{-}\;if\;\dot{\theta }<0 \end{array}\right.$$

**Figure 2:**
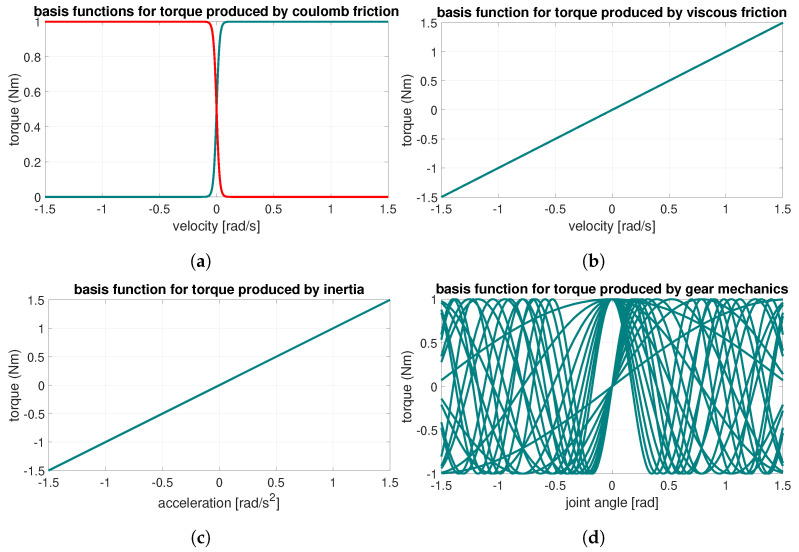
Torques due to different sources in a motor. (**a**) Coulomb friction (positive rotation in green, negative rotation in red). (**b**) Viscous friction. (**c**) Torque in a cobot joint produced by inertia deployed from Equation (11). (**d**) Torque in a cobot joint produced by a gearbox (harmonic drive) that is deployed from Equation (12).

For the Coulomb friction model described in Equation (8), we use a sigmoid representation of the basis function as follows:

(9) $$\left\{\begin{array}{c} \phi_{c}^{+}\left(x\right)=\frac{1}{1+\exp(-\alpha \dot{\theta })}\\ \phi_{c}^{-}\left(x\right)=\frac{1}{1+\exp(\alpha \dot{\theta })} \end{array}\right.,$$

where α>0 is a tuning parameter to adjust the shape of the curve near the zero velocity region and is chosen heuristically. The basis functions chosen as above yield a frictional curve as shown in Figure 2a.

Additionally, the basis function for the viscous friction (Figure 2b) can be written as:

(10) $$\phi_{v}\left(x\right)=\dot{\theta }$$

Note that all the coefficients $w_{c}^{+}$, $w_{c}^{-}$ and $w_{v}$ must be determined experimentally.

*(b)*  *Inertia*

The inertial torque measured by the torque sensor can be expressed as:

(11) $$\tau_{i}=\phi_{i}w_{i}=\ddot{\theta }I$$

where *I* represents the lumped inertia (Figure 2c) of the system. Torque due to inertia varies proportionally to the angular acceleration of the joint. For this reason, the basis function is chosen as $\phi_{i}\left(x\right)=\ddot{\theta }$ and the coefficient as $w_{i}=I$. The inertia of the system may also be experimentally estimated; however, to obtain this from the actuator data-sheet is also possible.

*(c)*  *Ripple torques from harmonic drive*

In harmonic drives when the flexspline undergoes periodic deformation, a ripple torque $\tau_{r}$ is generated, which is measured by the torque sensor at the joint. These ripples are periodic in nature with respect to the angle of rotation. Therefore, we consider *n* harmonics of sine and cosine signals as the basis functions, resulting in the ripple torque defined as:

(12) $$\begin{array}{cc} \tau_{r} & =\left[\cos\left(\theta \right),\ldots ,\cos\left(n\theta \right)\right]{\left[w_{c1},\ldots ,w_{cn}\right]}^{T}\\ \; & +\left[\sin\left(\theta \right),\ldots ,\sin\left(n\theta \right)\right]{\left[w_{s1},\ldots ,w_{sn}\right]}^{T}. \end{array}$$

(13) $$\begin{array}{cc} \tau_{r} & =\phi_{\cos}{\left(\theta \right)}^{T}w_{c}+\phi_{\sin}{\left(\theta \right)}^{T}w_{s} \end{array}$$

where $\phi_{\cos}\left(\theta \right)={\left[\cos\left(\theta \right),\ldots ,\cos\left(n\theta \right)\right]}^{T}$, $\phi_{\sin}\left(\theta \right)={\left[\sin\left(\theta \right),\ldots ,\sin\left(n\theta \right)\right]}^{T}$ are the collection of basis functions for the harmonics and $w_{c}={\left[w_{c1},\ldots ,w_{cn}\right]}^{T}$, $w_{s}={\left[w_{s1},\ldots ,w_{sn}\right]}^{T}$ the corresponding weights. The ripple torque in Equation (12) is plotted in Figure 2d where the torque values are represented by harmonic oscillation curves between −1 and 1 Nm.

*(d)*  *Torque offset*

Improper calibration of the torque sensor also can lead to erroneous readings. To account for this factor, a basis function to account for the offset in the sensed torque is introduced as follows:

(14) $$\tau_{o}=w_{o}$$

where the basis function is simply $\phi_{o}=1$. In summary, the torque sensed at every joint is modeled as a sum of the aforementioned torques as:

(15) $$\begin{array}{cc} \tau & =\tau_{f}+\tau_{i}+\tau_{r}+\tau_{o} \end{array}$$

(16) $$\begin{array}{cc} \tau & =\left[\phi_{v}\;\phi_{c}^{+}\;\phi_{c}^{-}\;\phi_{i}\;\phi_{\cos}{\left(\theta \right)}^{T}\;\phi_{\sin}{\left(\theta \right)}^{T}\;\phi_{o}\right]{\left[w_{v}\;w_{c}^{+}\;w_{c}^{-}\;w_{i}\;w_{c}^{T}\;w_{s}^{T}\;w_{o}\right]}^{T} \end{array}$$

All the dynamic parameters $w={\left[w_{v}\;w_{c}^{+}\;w_{c}^{-}\;w_{i}\;w_{c}^{T}\;w_{s}^{T}\;w_{o}\right]}^{T}$ are to be determined experimentally using a training dataset acquired when the robot joint is rotating without any external interaction.

## 3. Experimental Validation of Intrinsic Dynamics Compensation

The proposed framework is well suited for industrial wheel-bearing quality inspection as a good bearing is identified from a faulty one based on the frictional forced offered during its rotation. Currently, this kind of task is performed by human operators on the factory floors where the bearing is rotated manually and based on the frictional torque sensed by the human the quality of the bearing is determined. Typically, bearings with frictional torque above a threshold value are categorized as faulty ones and are rejected in the factory line. Automating this process with an off-the-shelf robot arm (with integrated joint torque sensors) poses the risk of wrongly disqualifying the product as the sensed torque also reads the intrinsic dynamics of the robot in addition to the actual frictional torques offered by the bearing. Therefore, compensating for the intrinsic dynamics leads to a more accurate estimation of the bearing frictional torques, which brings down the chances of wrongly disqualifying the bearing. With a detailed experiment analysis, we demonstrate how the intrinsic compensation minimizes the error in the frictional torque estimation.

For this experiment, the robot was equipped with a Robotiq 2F-85 parallel jaw gripper (Robotiq, Lévis, QC, Canada) to which the adapters for grasping the bearing were attached. A good-quality bearing was fastened onto a fixed platform equipped with a load cell to record the ground-truth/reference reaction torque measurements from the bearing.

### 3.1. Experimental Protocol

The first experiment is conducted to regress the intrinsic dynamic parameters under no-load conditions, using the model presented in Section 2.1. Once the model for robot intrinsic dynamics is known, we can use it to predict the intrinsics and subtract them from the torque readings to identify interaction torques, i.e., torques due to wheel-bearing friction. We then proceed with the second experiment, where the automated bearing inspection is performed by actuating only the end-effector joint of the robot.

#### 3.1.1. No-Load Conditions

To identify the intrinsic dynamics of the joint, the robot is initially configured to be in the zero position before the experiment was commenced. A trapezoidal torque profile of 30 s duration, with varying maximum values of 1–3 Nm was applied to the end-effector joint alone with alternating directions.

**Figure 3:**
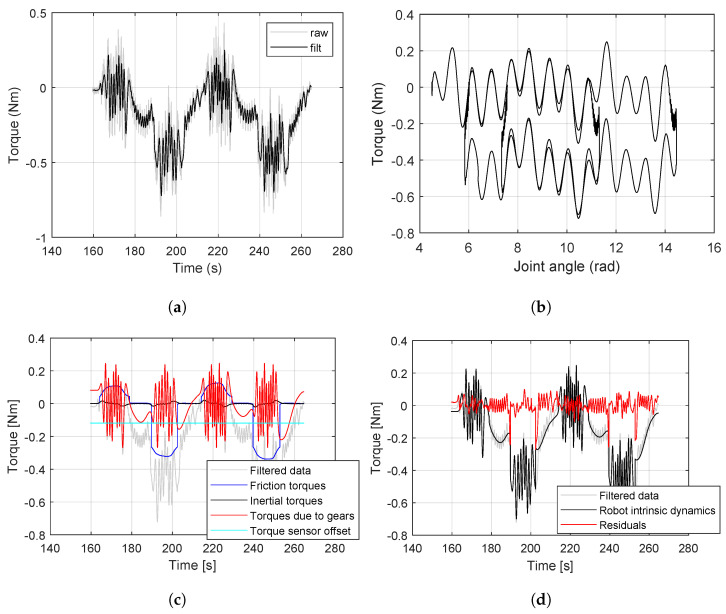
Intrinsic dynamic compensation from torque measurements under no-load conditions. (**a**) The 1 Hz-filtered torque of freely rotating joint. (**b**) Torque vs. joint angle is repeatable and directional. (**c**) Intrinsic dynamics components in Equations (7)–(14). (**d**) Residuals after intrinsic dynamic compensation approach zero.

The resulting sensed torque (from 7th joint torque sensor) is shown in Figure 3. In this scenario, the torque is filtered with a low pass filter with a cut-off frequency of 1 Hz to eliminate the noises in the torque reading. From the plot (Figure 3a), it can be observed that the integrated joint torque sensor, which ideally should read zero under no-load, instead records the torques due to the intrinsic dynamics of the joint. Additionally, over the joint angle, the measured torques are wave signals about −0.3 Nm away from the zero-axis. Upon plotting the sensed torque with regard to the joint angle (Figure 3b), the repeatable nature of this relationship can be viewed clearly. Specifically, the torque profiles are identical with regard to the joint angle, but shifted up or down based on a clockwise or anti-clockwise rotation of the joint.

The components of the intrinsic dynamics torque after identification are represented in Figure 3c. The two most stable components are the inertia $\tau_{i}\approx 0$ Nm and the torque offset $\tau_{o}=-0.1189$ Nm. The remaining components have larger variations in the torque values. The position-dependent torque ($\tau_{r}$ due to gear ripple) fluctuates around the zero-axis with an amplitude of ±0.2 Nm. The friction torque $\tau_{f}$ varies depending on the speed at which the joint rotated and had a minimum value of −0.3 Nm and a maximum value of +0.1 Nm. After identifying the intrinsic dynamics for the joint, we fit the sensed torque with intrinsic model prediction. Clearly, the residual measurements obtained after compensating the sensed torque by the predicted intrinsic dynamics, i.e., by subtracting the intrinsic dynamics from sensed torques, are centered around the zero-axis with smaller deviations (see Figure 3d). This is also an expected outcome ideally for the bearing inspection.

#### 3.1.2. Measuring Wheel-Bearing Torques

**Figure 4:**
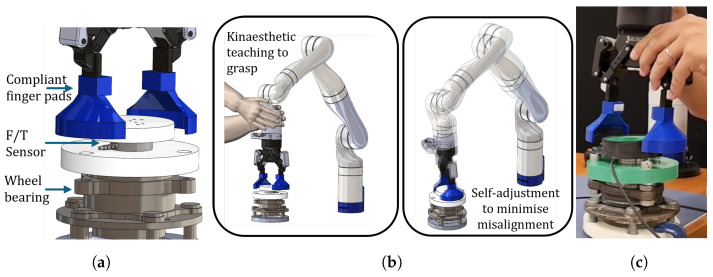
The design of compliant fingerpads is key to enabling the equivalent of a power grasp (without the need for complex articulation) of the wheel-bearing by conforming to its shape and preventing slipping while turning. The robot is guided by kinesthetic teaching under high compliance to grasp the wheel-bearing and is allowed to self-adjust to minimize any misalignment between robot and wheel-bearing axes. Once settled, the end-effector joint is driven in torque to assess the wheel-bearing friction. (**a**) Grasping the bearing. (**b**) High compliance mode. (**c**) Experiment.

We use a high-quality bearing from Schaeffler group to conduct the bearing inspection. To engage the wheel-bearing and transmit the axial torque generated by the wheel-bearing reliably, it is important for the robot’s end-effector axis to be aligned with the wheel-bearing along with a secure grasp to be established between the robot’s end-effector and the wheel-bearing flange. The shape of the wheel-bearing poses a challenge for this if we use the traditional parallel jaw gripper. For this reason, a flexible gripper adapter (see Figure 4a) to conform to the shape of the wheel-bearing was designed, prototyped and attached to each robot’s finger. The objective of this adapter is to mate with the contour of the wheel-bearing flange as the robot attempts to align it and secure the grasp. The bearing with its one ending surface being locked on a jig is placed on the same plane where the robot base is mounted.

The robot was operated with gravity compensation and one technician manually guided the robot to reach a suitable position for grasping the bearing. Once the position was decided, the gripper with the compliant finger pads was controlled to grasp the other adapters (to which the load cell was connected) on the bearing. With the robot in high compliance mode, it is allowed to self-align (Figure 4b) with the axis of rotation of the bearing. Subsequently, all the joints except the 7th joint (i.e., the end effector joint) were switched into impedance control mode with a high stiffness to maintain the respective positions (experimental setup shown in Figure 4c). A trapezoidal torque profile was applied to the joint to complete two revolutions in both clockwise and anti-clockwise directions alternately. The kinematic parameters along with the joint torque sensor readings were logged along with the data from the external load cell.

## 4. Results

The position, velocity and acceleration data collected from the joint 7 encoder were used to compute the intrinsic dynamics, which upon subtracting from the sensed torque isolated bearing torque. The obtained bearing torque was then compared against the torque read by the external load cell (that would not sense the intrinsic dynamics of the robot), which we consider as the ground truth.

### 4.1. Validation with External Load Cell

The external load cell, ATI F/T mini40 sensor (ATI, Markham, ON, Canada) is mounted on the load side (i.e., the inner ring of the bearing). To that end, a customized adapter that transmits the torque from the robot to the bearing through the load cell was prototyped. As shown in Figure 4c, the adapter comprised two cylindrical parts with different sizes to clamp the load cell and the outer ring of the bearing.

**Figure 5:**
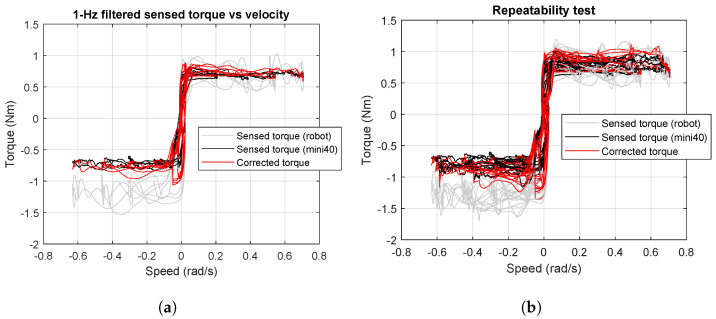
Illustration of the sensed torques in the benchmark ATI F/T sensor (mini40), and the Kinova robot arm in the instances with and without compensating the robot intrinsic dynamics. (**a**) Single-trial observations. (**b**) Observations over three trials.

In reference to Figure 5, the root mean square (RMS) errors between the joint torque sensor data and the load cell before and after the intrinsic dynamics compensation were found to be 27% and 11%, respectively. Specifically, the robot torque sensor feedback compared to the FT sensor torque shows a large deviation. Once our intrinsic dynamic model compensation is applied, this error decreases.

### 4.2. Repeatability of Proposed Compensation

In this section, we demonstrate the repeatability of the proposed intrinsic dynamics compensation. Here, the experiment protocol is same as the previous section. A total of three trials were conducted and we were able to successfully characterize the bearing in all the instances. The results are plotted in Figure 5b. Over the velocity, the maximum deviation between the reference torque of the wheel-bearing (measured by the external mini40 load cell) and the original robot sensor reading is roughly −0.7 Nm. Such a large deviation would have wrongly classified many good or qualified wheel-bearings as faulty and potentially eliminated them from the production line since wheel-bearing inspection requires strict precision. Equipped with our proposed method for compensating robot intrinsic dynamics, the corrected torque matched that measured by the external load cell (ATI mini40). Our dynamics intrinsic compensation thus can enhance the reliability and precision of bearing inspection by the robot arm while being repeatable enough over multiple experiments.

## 5. Conclusions

In this paper, intrinsic dynamics in the robot arm is identified and predicted via linear regression with a set of non-linear radial basis functions. Two experimental studies were conducted to validate the proposed approach. In the first experiment, the no-load rotation of the 7th joint (i.e., the end-effector joint) of the Kinova Gen3 robot was performed and the measurements from its joint torque sensor were used to identify the intrinsic dynamics of the robot. In the second experiment, the robot performed inspection of the bearing by rotating it with the end-effector joint and the bearing frictional torques were isolated after intrinsic dynamic compensation. To benchmark the obtained results, an external force/torque sensor was employed, which was set up on the bearing side to only measure the torques due to the wheel-bearing rotation. The results showed that the bearing torque measured by the joint 7th torque sensor before compensating intrinsic dynamics has an RMS error of 27%. After compensating using our intrinsic dynamic torque, the RMS error is in the range of 11%, and this result is repeatable enough across multiple experiments. Therefore, this study is meaningful for reducing the error in torque measurement of the robot joint and the fault rate in bearing inspection.

## Data Availability

Data and code available at https://researchdata.ntu.edu.sg/privateurl.xhtml?token=89aff4f4-e10f-4f24-a191-a5b967cfa250 (accessed on 20 November 2024).
